# The Quality and Reliability of Short Videos About Melasma on TikTok and Bilibili: A Cross‐Sectional Study

**DOI:** 10.1111/jocd.70578

**Published:** 2025-12-09

**Authors:** Qingyu Chen, Zhixuan Zhang, Yun Huang, Ziyue Wang

**Affiliations:** ^1^ Department of Neurology The Second Affiliated Hospital and Yuying Children's Hospital of Wenzhou Medical University Wenzhou China; ^2^ Department of International Medical Center The First People's Hospital of Foshan Foshan China; ^3^ School of Nursing and Midwifery University of Galway Galway Ireland

**Keywords:** Bilibili, health information quality, melasma, short‐video platforms, TikTok

## Abstract

**Background:**

Melasma is a common chronic hyperpigmentation disorder that substantially impairs patients' quality of life. With the rapid growth of short‐video platforms such as TikTok and Bilibili, an increasing number of patients are turning to these media for health‐related information. This study aimed to evaluate the quality and reliability of melasma‐related videos available on TikTok and Bilibili.

**Methods:**

Between August 17 and 19, 2025, we searched Douyin (the Chinese version of TikTok) and Bilibili using the Chinese keyword “黄褐斑” (“melasma”), and included the top 150 videos under each platform's default comprehensive ranking. The search and analysis were conducted in Chinese, reflecting the linguistic and geographical context of mainland China. Video characteristics and engagement metrics were recorded. The quality and reliability of the videos were independently evaluated by two researchers using the Global Quality Score (GQS) and the modified DISCERN (mDISCERN) instrument.

**Results:**

A total of 237 videos were included in this study. Content was dominated by clinical manifestations (46.8%), etiology (44.3%), and diagnosis (40.1%), whereas treatment‐related content was markedly underrepresented (9.7%). The median video length was 127.00 s (70.75–270.50) on Bilibili and 47.00 s (35.00–96.00) on TikTok. TikTok videos achieved significantly higher engagement than Bilibili (*p* < 0.05). Overall video quality was moderate, with both GQS and mDISCERN showing a median score of 3.00 (IQR: 2.00–4.00). The mDISCERN score of Bilibili videos was 3.00 (3.00–4.00), significantly higher than TikTok (*p* < 0.05). Videos uploaded by healthcare professionals scored 3.00 (3.00–4.00) on GQS and 3.00 (2.00–4.00) on mDISCERN, both significantly higher than those uploaded by non‐healthcare professionals (*p* < 0.05).

**Conclusions:**

This study found that melasma‐related short videos presented an incomplete content structure, with treatment‐related information being markedly underrepresented. The overall quality of the videos was moderate, whereas those produced by healthcare professionals demonstrated higher quality and reliability. Future efforts should encourage greater participation from healthcare professionals and the implementation of refined content strategies, with the aim of improving both the quality and educational value of dermatology‐related short video resources.

## 1 Introduction

Melasma is a common acquired hyperpigmentation disorder characterized by symmetrical brown or gray‐brown macules and patches, predominantly affecting sun‐exposed areas of the face [1]. It is particularly prevalent in women of reproductive age and is strongly associated with hormonal fluctuations, ultraviolet radiation, and genetic predisposition [2]. Although melasma is not a life‐threatening disease, its visibility on the face often results in considerable cosmetic concerns and psychological distress, including reduced self‐confidence, anxiety, and social withdrawal [3]. Several studies have reported prevalence rates ranging from 1.5% to 33% worldwide, with the highest rates observed in Asian and Latin American populations, where cultural emphasis on facial appearance further magnifies the psychosocial burden of the disease [4, 5]. Given its chronic and recurrent nature, patients frequently seek ongoing medical guidance regarding etiology, preventive measures, and effective treatment strategies.

In parallel with the rising demand for health‐related information, short‐video platforms such as TikTok and Bilibili have rapidly emerged as major channels for public health communication [6]. TikTok, known for its algorithm‐driven recommendations, has become one of the most widely used platforms among young adults globally, while Bilibili is particularly popular among younger Chinese users for its longer and more detailed educational content [7]. The interactive nature of these platforms, combined with their wide reach and audiovisual presentation, makes them uniquely suited for disseminating medical knowledge. Nevertheless, the information provided on such platforms is largely user‐generated and lacks standardized review or regulation. Consequently, the quality of health‐related videos is highly variable, raising concerns about misinformation, biased content, and the potential for misleading audiences [8, 9]. For conditions like melasma, where patients are often motivated to self‐manage and seek cosmetic solutions, the quality and reliability of online information are especially critical.

Previous research has begun to address the quality of short‐video health information for a variety of medical conditions [10, 11]. Studies have analyzed TikTok videos related to ophthalmology, cardiovascular diseases, dermatological disorders, and endocrinological conditions, consistently finding substantial variation in accuracy, reliability, and educational value [12, 13, 14]. For example, evaluations of cataract‐related videos revealed that while some content offered useful explanations, the majority were of only moderate quality [15]. Similarly, analyses of hypertension videos indicated that professional contributions were limited and that therapeutic guidance was particularly underrepresented [16]. These findings suggest that despite the promise of short‐video platforms for health education, the actual quality of content available to the public is often inadequate [17, 18, 19]. However, no study has evaluated the content quality and reliability of melasma‐related videos on short‐video platforms to date. Considering the high prevalence of melasma and its significant psychosocial impact, this represents a noteworthy gap in current research.

Therefore, the purpose of this study is to evaluate the content, quality, and reliability of melasma‐related videos on TikTok and Bilibili, with the aim of identifying current deficiencies and guiding future efforts to improve the dissemination of high‐quality melasma‐related information on short‐video platforms.

## 2 Methods

### 2.1 Data Collection

**FIGURE 1:**
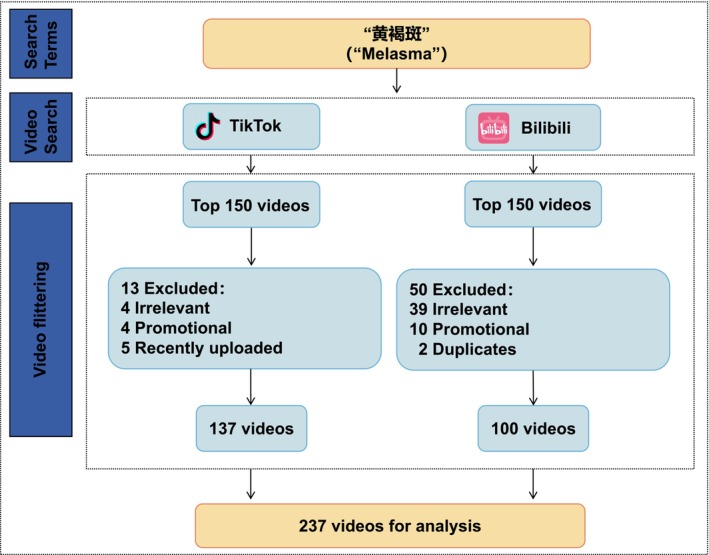
Flow diagram of video selection and exclusion process.

This cross‐sectional study was conducted on two widely used short‐video platforms in China, TikTok and Bilibili. A systematic search was performed between August 17 and August 19, 2025, using the keyword “黄褐斑” (“melasma”). For each platform, the top 150 most relevant videos returned by the default search algorithm were retrieved, yielding an initial pool of 300 videos. After screening, 237 videos that met the eligibility criteria were included in the final analysis. The selection process is illustrated in Figure 1.

### 2.2 Inclusion and Exclusion Criteria

Videos were eligible if they were in Chinese, directly addressed melasma, and provided health‐related information such as etiology, clinical manifestations, diagnosis, treatment, or prevention. Exclusion criteria included: (1) duplicate videos; (2) videos irrelevant to melasma; (3) commercial advertisements; (4) videos uploaded within 1 week prior to data collection, in order to minimize the potential instability of engagement metrics.

### 2.3 Video Characteristics

For each included video, general features were extracted, including video duration (seconds), number of likes, comments, collections, and shares. Data were collected on the same day to reduce variability introduced by continuous user interaction.

### 2.4 Content Classification

The content of each video was classified into six categories, namely epidemiology, etiology, clinical manifestations, diagnosis, treatment, and prevention, following previously published frameworks [20]. A single video could be assigned to more than one category if it covered multiple aspects. An example of a representative video from the “etiology” category is provided in Supporting Information S1 to illustrate the typical content and presentation style.

### 2.5 Quality Assessment

Video quality was independently assessed by two trained researchers with undergraduate education in medical‐related fields using two validated instruments: the Global Quality Score (GQS) and the modified DISCERN (mDISCERN). The GQS is a five‐point Likert scale that evaluates the overall quality and educational value of health information, while the mDISCERN consists of five items assessing reliability [21, 22]. The detailed scoring criteria are presented in Tables 1 and 2. When discrepancies arose between the two researchers' ratings, the final decision was resolved through discussion with a third researcher who has clinical experience in dermatology and supervised the evaluation process.

**TABLE 1:** Global Quality Score (GQS) scale used for video quality assessment.

Item features	Points
Poor quality; poor flow of the videos; most information missing; not at all useful for patients	1
Generally poor quality; some information listed, but many important topics missing; of very limited use to patients	2
Moderate quality; suboptimal flow; some important adequately discussed, but other information poorly discussed; somewhat useful for patients	3
Good quality and generally good flow; most of the relevant information listed, but some topics not covered; useful for patients	4
Excellent quality and flow; very useful for patients	5

**TABLE 2:** Modified DISCERN (mDISCERN) tool used for evaluation of reliability.

Reliability score
1. Is the video clear, concise, and understandable?
2. Are valid sources cited?
3. Is the content presented balanced and unbiased?
4. Are additional sources of content listed for patient reference?
5. Are areas of uncertainty mentioned?

### 2.6 Uploader Classification

Uploaders were first dichotomized into professionals (specialized and non‐specialized physicians and professional institutions) and non‐professionals (non‐professional institutions and individual users). Specialized physicians referred to board‐certified dermatologists or physicians with dermatology expertise, while non‐specialized physicians were defined as medical staff without formal dermatology specialization. Professional institutions included hospitals, universities, and academic organizations, whereas non‐professional institutions referred to entities such as beauty salons and commercial media platforms. Individual users referred to laypersons without medical training. For further analyses, uploaders were categorized into four groups: specialized physicians, non‐specialized physicians, institutions, and individual users. Although institutions were classified as professional or non‐professional according to their medical affiliation, these subgroups were merged into a single “institution” category for subsequent analyses due to limited sample size and conceptual comparability.

### 2.7 Statistical Analysis

Normality of continuous variables was tested using the Shapiro–Wilk test. Variables with a normal distribution were expressed as mean ± standard deviation, while non‐normally distributed variables were summarized as medians with interquartile ranges (IQRs). Differences between two groups were assessed using the Mann–Whitney *U* test, and comparisons among three or more groups were performed with the Kruskal–Wallis test followed by Dunn's post hoc pairwise comparisons when appropriate. A two‐sided *p* value < 0.05 was considered statistically significant. Data processing, statistical analyses, and visualization were conducted using R version 4.3.3.

### 2.8 Artificial Intelligence Tools Statement

No generative artificial intelligence (AI) or large language model (LLM) tools were used in the preparation of this manuscript.

## 3 Results

### 3.1 General Characteristics, Content Distribution, and Overall Quality of Videos

A total of 237 short videos related to melasma were included. The median video duration was 76 s (43–184), ranging from 12 to 629 s. The included videos received a median of 930 likes (73–5547), 686 collections (88–3689), 94 comments (11–503), and 219 shares (23–1484). The distribution of engagement was highly skewed, with the number of likes ranging from 11 to 20 543, collections from 2 to 12 487, comments from 1 to 2356, and shares from 0 to 6879. In terms of content, clinical manifestations (46.8%), etiology (44.3%), and diagnosis (40.1%) were the most common, followed by prevention (35.4%) and epidemiology (17.7%), while treatment was the least represented (9.7%). Video quality and reliability were generally moderate, with both GQS and mDISCERN median scores at 3 (IQR 2–4), indicating most videos were of intermediate quality (Table 3).

**TABLE 3:** General characteristics of included videos (*n* = 237). Values are shown as median (IQR).

Variables	Total (*n* = 237)
General information	
Video length, M (Q_1_, Q_3_)	76.00 (43.00, 184.00)
Likes, M (Q_1_, Q_3_)	930.00 (73.00, 5547.00)
Collections, M (Q_1_, Q_3_)	686.00 (88.00, 3689.00)
Comments, M (Q_1_, Q_3_)	94.00 (11.00, 503.00)
Shares, M (Q_1_, Q_3_)	219.00 (22.50, 1484.00)
Video content (*n*) (%)	
Epidemiology	42 (17.72%)
Etiology	105 (44.30%)
Clinical manifestations	111 (46.84%)
Diagnosis	95 (40.08%)
Treatment	23 (9.70%)
Prevention	84 (35.44%)
Video quality	
GQS score, M (Q_1_, Q_3_)	3.00 (2.00, 4.00)
mDISCERN score, M (Q_1_, Q_3_)	3.00 (2.00, 4.00)

Abbreviations: GQS, Global Quality Score; IQR, interquartile range; M, Median; mDISCERN, modified DISCERN; Q_1_, 1st Quartile; Q_3_, 3rd Quartile.

### 3.2 Comparison of Video Characteristics and Quality Between TikTok and Bilibili

**FIGURE 2:**
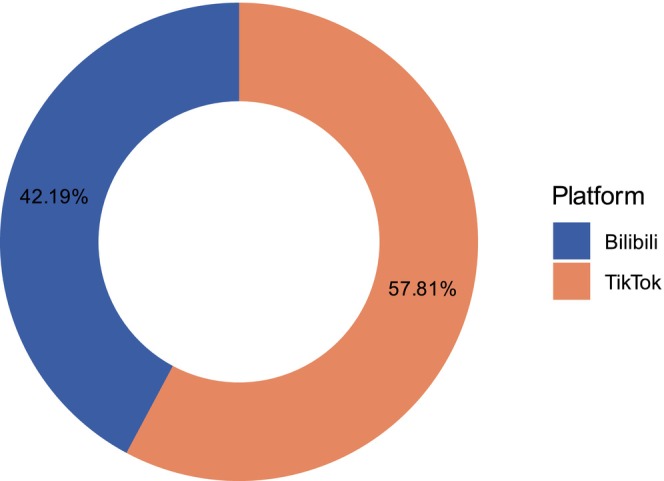
Distribution (proportion) of videos across TikTok and Bilibili platforms.

**FIGURE 3:**
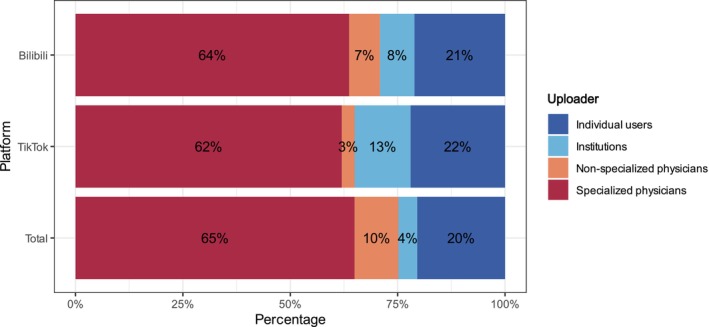
Distribution (proportion) of uploader identities across TikTok and Bilibili platforms.

**FIGURE 4:**
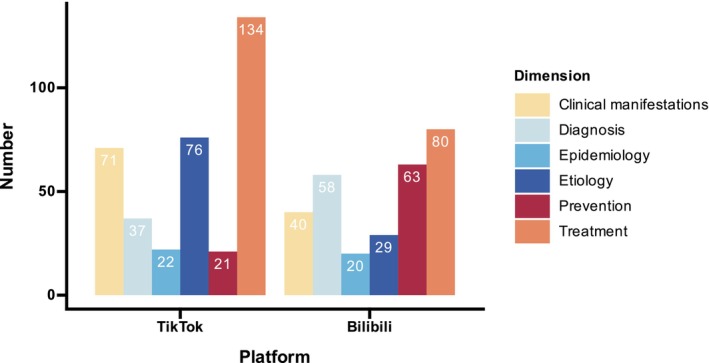
Distribution of melasma‐related video content categories (epidemiology, etiology, clinical manifestations, diagnosis, treatment, and prevention) across TikTok and Bilibili platforms.

Regarding platform distribution, TikTok contributed 137 videos (57.8%), while Bilibili provided 100 (42.2%) (Figure 2). The composition of uploader identities also differed: TikTok was dominated by specialized physicians and individual users, while Bilibili had a higher proportion of institutional uploads (Figure 3). Significant differences were observed in video characteristics and engagement metrics. TikTok videos were shorter, with a median duration of 47 s (35–96), compared with 127 s (71–271) on Bilibili (*p <* 0.001). Engagement metrics were markedly higher on TikTok, with a median of 4381 likes (1352–11 846) versus 60.5 (7–194), 2941 collections (835–7775) versus 86 (5–206), 359 comments (125–1085) versus 9 (1–31), and 1076 shares (345–3639) versus 21 (1–61), all *p <* 0.001. In terms of quality, TikTok had higher GQS scores (median 3.0, IQR 3.0–4.0), while Bilibili had superior mDISCERN scores (median 3.0, IQR 3.0–4.0 vs. 2.0–3.0, *p <* 0.001) (Table 4). Content distribution also varied: both platforms emphasized clinical manifestations, etiology, and diagnosis, but TikTok had lower proportions of prevention (27.7% vs. 45.0%) and treatment (7.3% vs. 12.0%), while Bilibili showed higher representation in diagnosis (45.0% vs. 36.5%) and epidemiology (24.0% vs. 13.1%) (Figure 4).

**TABLE 4:** Comparison of video characteristics and quality scores between TikTok and Bilibili.

Variables	Bilibili (*n* = 100)	TikTok (*n* = 137)	Statistic	*p*
Video length, M (Q_1_, Q_3_)	127.00 (70.75, 270.50)	47.00 (35.00, 96.00)	*Z* = −7.29	< 0.001
Likes, M (Q_1_, Q_3_)	60.50 (6.75, 193.75)	4381.00 (1352.00, 11 846.00)	*Z* = −12.04	< 0.001
Collections, M (Q_1_, Q_3_)	86.00 (4.50, 206.00)	2941.00 (835.00, 7775.00)	*Z* = −10.67	< 0.001
Comments, M (Q_1_, Q_3_)	9.00 (1.00, 30.75)	359.00 (125.00, 1085.00)	*Z* = −11.04	< 0.001
Shares, M (Q_1_, Q_3_)	21.00 (1.00, 60.50)	1076.00 (345.00, 3639.00)	*Z* = −11.40	< 0.001
GQS, M (Q_1_, Q_3_)	3.00 (2.00, 3.00)	3.00 (3.00, 4.00)	*Z* = −6.41	< 0.001
Mdiscern, M (Q_1_, Q_3_)	3.00 (3.00, 4.00)	3.00 (2.00, 3.00)	*Z* = −4.40	< 0.001

Abbreviations: GQS, Global Quality Score; IQR, interquartile range; M, Median; mDISCERN, modified DISCERN; *p*, *p*‐value; Q_1_, 1st Quartile; Q_3_, 3rd Quartile.

### 3.3 Comparison of Video Characteristics and Quality Across Different Uploader Sources

**FIGURE 5:**
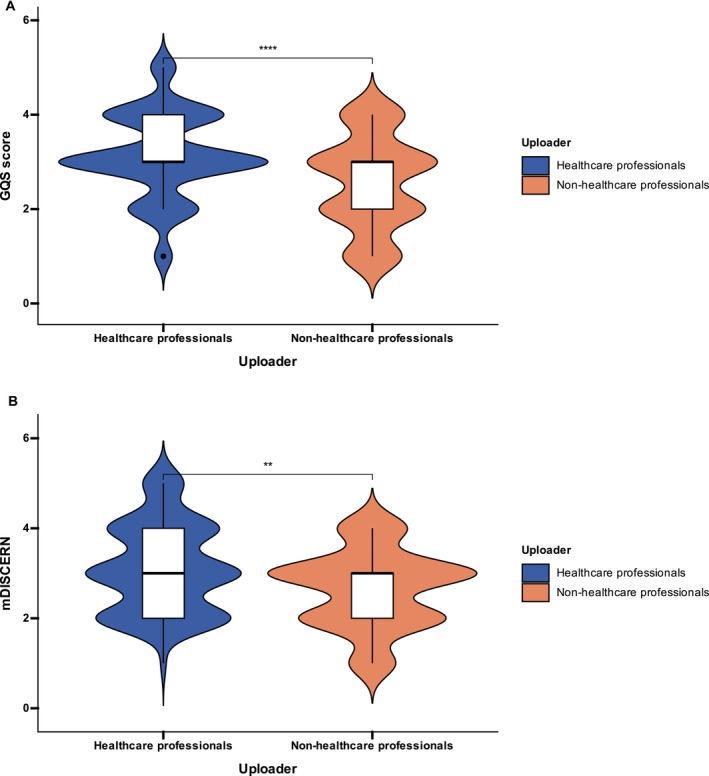
Overall quality scores by uploader professional status. *Note*: ** : *p* < 0.01, **** : *p* < 0.0001.

In the binary comparison (Table 5), professionals (specialized and non‐specialized physicians) accounted for 74.3% of the videos, while non‐professionals (institutions and individual users) accounted for 25.7%. Videos uploaded by non‐professionals were significantly longer, with a median duration of 185 s (91–266), compared with 59 s (39–108) for professionals (*p <* 0.001). In contrast, engagement metrics including likes, collections, comments, and shares did not differ significantly between the two groups (all *p* > 0.05). Regarding quality, Figure 5A shows that professionals achieved significantly higher GQS scores than non‐professionals, with a median of 3.0 (3.0–4.0) versus 3.0 (2.0–3.0) (*p* < 0.001). Figure 5B shows consistent results for mDISCERN, where professionals again scored higher at 3.0 (3.0–4.0) compared with 2.0 (2.0–3.0) for non‐professionals (*p* = 0.004).

**TABLE 5:** Comparison of video characteristics and quality scores between healthcare professionals and non‐professionals.

Variables	Total (*n* = 237)	Healthcare professionals (*n* = 176)	Non‐healthcare professionals (*n* = 61)	Statistic	*p*
Video length, M (Q_1_, Q_3_)	76.00 (43.00, 184.00)	59.00 (39.00, 107.75)	185.00 (91.00, 266.00)	*Z* = −6.12	< 0.001
Likes, M (Q_1_, Q_3_)	930.00 (73.00, 5547.00)	1004.00 (72.75, 6001.50)	670.00 (80.00, 2227.00)	*Z* = −1.18	0.237
Collections, M (Q_1_, Q_3_)	686.00 (88.00, 3689.00)	780.50 (90.00, 4785.50)	368.00 (88.00, 1359.00)	*Z* = −1.87	0.062
Comments, M (Q_1_, Q_3_)	94.00 (11.00, 503.00)	88.50 (9.00, 366.75)	113.00 (12.00, 891.00)	*Z* = −1.50	0.134
Shares, M (Q_1_, Q_3_)	219.00 (22.50, 1484.00)	318.00 (21.00, 2044.00)	173.50 (32.75, 633.75)	*Z* = −1.36	0.175
GQS, M (Q_1_, Q_3_)	3.00 (2.00, 4.00)	3.00 (3.00, 4.00)	3.00 (2.00, 3.00)	*Z* = −3.93	< 0.001
Mdiscern, M (Q_1_, Q_3_)	3.00 (2.00, 4.00)	3.00 (2.00, 4.00)	3.00 (2.00, 3.00)	*Z* = −2.89	0.004

Abbreviations: GQS, Global Quality Score; IQR, interquartile range; M, Median; mDISCERN, modified DISCERN; *p*, *p*‐value; Q_1_, 1st Quartile; Q_3_, 3rd Quartile.

**FIGURE 6:**
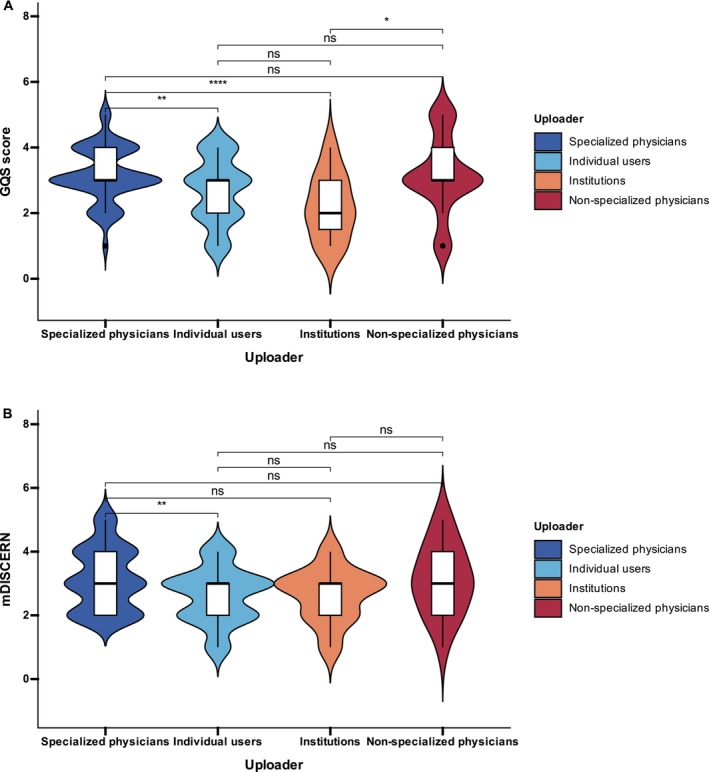
Overall quality scores of videos (Global Quality Score, GQS; modified DISCERN, mDISCERN) according to uploader categories (specialized physicians, non‐specialized physicians, institutions, and individual users). *Note:* ns: not significant, *p < 0.05, **p < 0.01, ****p < 0.0001.

In the four‐group comparison (Table 6), specialized physicians constituted the largest group (63.7%, *n* = 151), followed by individual users (21.1%), institutions (8.0%), and non‐specialized physicians (7.2%) (Figure 3). Regarding video duration, individual users uploaded the longest videos with a median of 189 s (94–294), which was significantly longer than those of specialized physicians (55 s, 37–98) and non‐specialized physicians (84 s, 52–163) (both *p <* 0.001), but not significantly different from institutions (152 s, 85–249) (*p >* 0.05). Institutions also had significantly longer videos than specialized physicians (*p <* 0.001), while non‐specialized physicians' videos were longer than those of specialized physicians (*p <* 0.01). Engagement metrics were highest in videos from non‐specialized physicians, with medians of 6018 likes (2154–13 284), 4235 collections (1589–9960), and 1740 shares (611–4238). These were significantly higher than those of specialized physicians and institutions (all *p <* 0.001), but not significantly different from those of individual users (*p >* 0.05). Individual users also achieved significantly higher engagement than institutions (all *p <* 0.001). For comments, no significant difference was observed between non‐specialized physicians and individual users (*p >* 0.05), but both groups had significantly more comments than specialized physicians and institutions (all *p <* 0.001). As for quality, Figure 6A shows that specialized physicians and institutions achieved the highest GQS scores, with medians of 3.0 (3.0–4.0), significantly higher than those of non‐specialized physicians (2.0, 2.0–3.0) and individual users (2.0, 2.0–3.0) (all *p <* 0.001). Individual users scored significantly higher than non‐specialized physicians (*p <* 0.05), while no significant difference was observed between specialized physicians and institutions (*p >* 0.05). Figure 6B shows the results for mDISCERN, which followed the same pattern. Specialized physicians and institutions had the highest scores (3.0, 3.0–4.0), significantly higher than those of non‐specialized physicians (2.0, 2.0–3.0) and individual users (2.0, 2.0–3.0) (all *p <* 0.001). Individual users again scored higher than non‐specialized physicians (*p <* 0.05), whereas specialized physicians and institutions did not differ significantly from each other (*p >* 0.05).

**TABLE 6:** Characteristics and quality scores of videos according to uploaders.

Variables	Total (*n* = 237)	Individual users (*n* = 50)	Institutions (*n* = 19)	Non‐specialized physicians (*n* = 17)	Specialized physicians (*n* = 151)	Statistic	*p*
Video length, M (Q_1_, Q_3_)	76.00 (43.00, 184.00)	189.00 (103.50, 265.50)	92.00 (58.00, 189.50)	60.00 (45.00, 200.00)	55.00 (37.00, 101.00)	*χ* ^2^ = 45.50#	< 0.001
Likes, M (Q_1_, Q_3_)	930.00 (73.00, 5547.00)	937.00 (112.50, 3332.75)	51.00 (3.00, 212.00)	2954.00 (757.00, 11 846.00)	1021.00 (73.50, 5988.50)	*χ* ^2^ = 20.15#	< 0.001
Collections, M (Q_1_, Q_3_)	686.00 (88.00, 3689.00)	793.00 (101.75, 1764.75)	59.00 (3.00, 149.00)	1768.00 (546.00, 7961.00)	828.00 (96.50, 4823.00)	*χ* ^2^ = 22.97#	< 0.001
Comments, M (Q_1_, Q_3_)	94.00 (11.00, 503.00)	218.50 (14.75, 1038.00)	6.00 (0.00, 42.00)	159.00 (67.00, 797.00)	86.00 (9.00, 397.50)	*χ* ^2^ = 15.22#	0.002
Shares, M (Q_1_, Q_3_)	219.00 (22.50, 1484.00)	222.00 (36.00, 757.00)	22.00 (0.00, 113.00)	772.00 (207.00, 4564.00)	275.00 (23.00, 2049.50)	*χ* ^2^ = 16.83#	< 0.001
GQS, M (Q_1_, Q_3_)	3.00 (2.00, 4.00)	3.00 (2.00, 3.00)	2.00 (1.50, 3.00)	3.00 (3.00, 4.00)	3.00 (3.00, 4.00)	*χ* ^2^ = 22.59#	< 0.001
Mdiscern, M (Q_1_, Q_3_)	3.00 (2.00, 4.00)	3.00 (2.00, 3.00)	3.00 (2.00, 3.00)	3.00 (2.00, 4.00)	3.00 (2.00, 4.00)	*χ* ^2^ = 10.86#	0.013

*Note:* The symbol “#” in Table 6 denotes that the Kruskal–Wallis (non‐parametric) test for the corresponding variable reached statistical significance.

Abbreviations: GQS, Global Quality Score; IQR, interquartile range; M, Median; mDISCERN, modified DISCERN; *p*, *p*‐value; Q_1_, 1st Quartile; Q_3_, 3rd Quartile.

### 3.4 Correlation Analysis Between Engagement Metrics and Quality Scores

**FIGURE 7:**
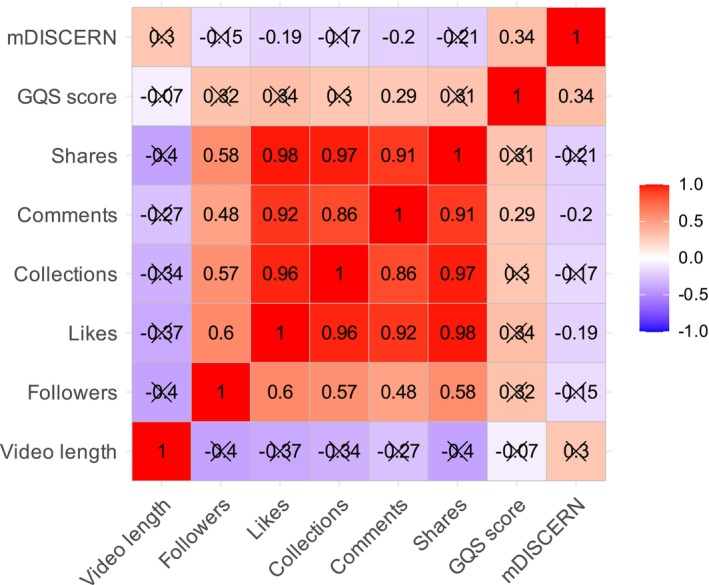
Correlation matrix of video length, engagement metrics (likes, collections, comments, shares), and quality scores (GQS and mDISCERN).

Engagement metrics were strongly and positively correlated with each other. Likes correlated with collections (*r* = 0.98), shares (*r* = 0.97), and comments (*r* = 0.91, all *p* < 0.001); collections correlated with shares (*r* = 0.97) and comments (*r* = 0.86, all *p* < 0.001). A moderate positive correlation was also observed between the number of followers and engagement metrics (*r* = 0.48–0.58, all *p* < 0.001). No significant associations were found between engagement metrics and quality scores (GQS or mDISCERN). A moderate positive correlation was observed between GQS and mDISCERN (*r* = 0.34, *p* < 0.001), indicating acceptable consistency between the two evaluation tools (Figure 7).

## 4 Discussion

Melasma is a common acquired pigmentary disorder, clinically characterized by symmetric brownish macules and patches on the face. It tends to follow a chronic course, is prone to recurrence, and is particularly prevalent among women. Because of its considerable impact on facial appearance, patients frequently experience anxiety and psychological distress [23]. Thus, the management of melasma involves not only dermatological interventions but also patient adherence and health literacy. In recent years, short‐video platforms have become increasingly important sources of medical information, with direct influence on patients' healthcare‐seeking behavior and treatment decisions. Against this backdrop, we systematically evaluated 237 melasma‐related short videos on TikTok and Bilibili, analyzing their content, quality, and reliability, with the goal of informing clinical practice in patient education and management.

### 4.1 Overall Characteristics and General Quality of Videos

In this study, the included short videos showed considerable variation in duration and engagement metrics. The median length was 76 s, ranging from just over 10 s to several minutes, reflecting the highly fragmented mode of information delivery on short‐video platforms. This pattern is consistent with previous analyses of acne‐related videos, which also highlighted the predominance of “short and fast‐paced” content in dermatology [24]. However, for melasma, a chronic condition with complex management requirements, excessively short duration may restrict the completeness and depth of information provided. With respect to engagement and quality, overall levels were moderate but unevenly distributed. While some videos accumulated tens of thousands of likes and thousands of shares, others received minimal attention, presenting a clear “Pareto effect.” This phenomenon is likely linked to platform recommendation algorithms, which amplify highly interactive content while potentially marginalizing more rigorous but less entertaining material. Further quality assessment revealed that the median GQS and mDISCERN scores were both 3, indicating limited educational value. This finding echoes prior studies of hypertension‐ and cataract‐related videos, suggesting that medical short videos, in general, remain suboptimal in reliability and scientific rigor.

### 4.2 Imbalance Between Content Distribution and Patient Needs

Our analysis further demonstrated that clinical manifestations (46.8%), etiology (44.3%), and diagnosis (40.1%) were the most frequently covered themes, while treatment‐related information was present in only 9.7% of videos. This imbalance is consistent with findings in other dermatological conditions. For example, evaluations of eczema and *Helicobacter pylori* videos highlighted a paucity of treatment‐related information, despite patients' strong demand for such content [25, 26]. For melasma, patients require clear and reliable guidance on efficacy, recurrence risk, adverse effects, and costs of treatment. The scarcity of therapeutic information therefore risks widening the gap between patient expectations and clinical realities, reducing adherence and satisfaction. In clinical practice, dermatologists often encounter patients who attempt self‐treatment based on video recommendations, sometimes leading to complications such as steroid‐induced dermatitis. Increasing the proportion of treatment‐focused educational content should thus be prioritized in future dermatological communication on short‐video platforms.

### 4.3 Platform Differences and Their Impact on Patient Perceptions

Significant differences were observed between TikTok and Bilibili. TikTok videos were shorter (median 47 s) but received higher engagement, whereas Bilibili videos were longer (median 127 s) and achieved higher mDISCERN scores, indicating better reliability. These differences reflect platform‐specific algorithms and user demographics. Consistent with prior research on systemic lupus erythematosus videos, TikTok tends to favor fragmented, entertainment‐oriented content, while Bilibili and YouTube are more conducive to evidence‐based presentations [27]. Such ecological differences shape patient perceptions: TikTok's rapid, visually stimulating style fosters expectations of “instant results,” whereas Bilibili's longer format facilitates explanations of long‐term, multimodal management strategies. Clinically, this divergence manifests when patients, influenced by short‐form content, express frustration with the gradual course of treatment. Dermatologists should therefore emphasize during consultations that most therapies for melasma require sustained use and may only produce incremental improvement, thereby correcting misconceptions propagated by platform‐driven narratives.

### 4.4 Uploader Identity and the Marginalization of Professional Voices

This study revealed that, overall, videos uploaded by professionals were of significantly higher quality than those uploaded by non‐professionals, highlighting the crucial role of medical background in ensuring reliability. However, such higher‐quality videos did not demonstrate advantages in engagement and were often surpassed by non‐professional content, reflecting the paradox of “high quality but low popularity.” Further analysis showed that among professional uploaders, videos from specialized physicians scored highest in quality, followed by non‐specialized physicians, whereas within non‐professional uploaders, videos from institutions and lay users were of generally lower quality but often attracted more comments and shares. This pattern is consistent with findings from other medical video analyses, underscoring the marginalization of professional voices in the short‐video ecosystem. Clinically, this means that patients exposed to non‐professional content may develop perceptions inconsistent with scientific evidence, thereby increasing the burden on physicians to correct misinformation during consultations. Although international studies suggest that platform features such as physician verification badges can modestly enhance credibility, their effect remains limited unless reinforced by recommendation algorithms. Therefore, dermatologists should not only continue producing evidence‐based content but also explore more engaging formats or collaborate with professional science communicators to enhance visibility, ensuring that reliable information is not overshadowed by less rigorous but more entertaining material [28].

### 4.5 Video Duration and Its Relationship to Quality

Our results revealed a moderate positive correlation between video duration and quality scores, particularly pronounced for mDISCERN, suggesting that longer videos tend to deliver more comprehensive, evidence‐based information and comparisons of treatment options. However, no significant association was found between duration and engagement, suggesting that video length alone did not determine popularity. This indicates that factors other than duration, such as recommendation algorithms and entertainment‐oriented formats, might play a greater role in shaping engagement patterns. While longer videos may allow for more comprehensive content, this does not necessarily translate into higher visibility or interaction. The heatmap analysis provided further insights: very short videos, while capable of capturing attention quickly, may generally lack sufficient educational value and convey fragmented information, whereas excessively long but poorly structured videos might also fail to sustain audience attention and could even reduce dissemination effectiveness due to possible informational overload [29]. Thus, duration alone is not necessarily the decisive factor; structural design and clarity of presentation are equally critical. For melasma, a chronic disorder requiring long‐term, multimodal management, the optimal approach may not be merely to extend duration but rather to optimize medium‐length videos through modular explanations, concise key‐point summaries, intuitive visuals, and on‐screen citation of references, thereby potentially increasing both informational density and comprehensibility. Such a strategy is consistent with prior findings in oncology and osteoarthritis video research, where modest extensions in duration combined with structured design were shown to improve completeness and educational value. Taken together, these findings suggest that future dermatology‐related short videos should focus on achieving a “dual balance of duration and structure” to maximize both educational quality and dissemination impact.

### 4.6 Clinical and Public Health Implications

Taken together, these findings highlight several challenges in melasma‐related health communication via short‐video platforms: the disconnect between popularity and quality, the scarcity of therapeutic guidance, the shaping of patient perceptions by platform ecology, the marginalization of professional voices, and the duration–quality relationship. Each has direct implications for clinical dermatology [30]. First, dermatologists should proactively ask whether patients have consulted online videos and provide corrective, evidence‐based guidance during consultations. Studies in other medical fields have shown that patient reliance on incorrect online content increases the burden of clinical communication and can undermine trust. Second, dermatological societies and healthcare institutions should assume greater responsibility for producing authoritative, patient‐centered short videos, particularly focusing on treatment and prevention, to fill existing gaps. Third, high‐quality short videos have the potential to improve adherence and outcomes: prior evidence indicates that when patients receive clearer educational information before treatment, their compliance and satisfaction increase significantly. Leveraging these insights could improve both individual patient care and broader public dermatological health literacy.

### 4.7 Limitations and Future Directions

Several limitations should be acknowledged. This study was cross‐sectional, capturing content at a single time point, whereas short‐video platforms evolve rapidly. Only Chinese‐language videos were included, limiting generalizability to other linguistic or cultural contexts. Although validated tools such as GQS and mDISCERN were employed, some subjectivity remains inherent to quality assessments. Furthermore, our search strategy focused on the top 150 algorithmically recommended videos per platform, which approximates user experience but may have excluded less visible high‐quality content. Future studies should adopt longitudinal designs to track changes over time and develop evaluation instruments tailored to the unique features of short‐video content. Incorporating patient perspectives into such evaluations would also provide a more comprehensive understanding of the impact of video content on decision‐making and adherence.

## 5 Conclusion

In summary, our study provides the first systematic assessment of melasma‐related short videos on TikTok and Bilibili. Although these videos achieved wide reach and high levels of engagement, their overall quality was only moderate, and treatment‐related information was markedly underrepresented. A clear discrepancy between popularity and reliability was observed, underscoring the risk that patients may be influenced more by appealing than by accurate content. Greater involvement of healthcare professionals, adoption of creative yet reliable communication strategies, and platform‐level mechanisms to highlight trustworthy sources are needed to enhance the value of short‐video platforms for dermatological health education. Strengthening these efforts has the potential to bridge current information gaps and better meet the needs of patients with melasma.

## Author Contributions

**Qingyu Chen:** investigation, methodology, writing – original draft. **Zhixuan Zhang:** data interpretation supervision. **Yun Huang:** conceptualization, project administration, writing – review and editing. **Ziyue Wang:** writing – review and editing, supervision, funding acquisition, formal analysis.

## Funding

This study was supported by the National College Student Innovation and Entrepreneurship Training Program (grant number 202310343061X) and the Zhejiang Province College Student Science and Technology Innovation Activity Plan (grant number 2024R413B080).

## Ethics Statement

This study exclusively used publicly available video content from Chinese platforms of TikTok and Bilibili. No clinical data, human specimens, animal experiments, or personal privacy information were involved in this study. Consequently, ethical review was not required for this research.

## Consent

The authors have nothing to report.

## Conflicts of Interest

The authors declare no conflicts of interest.

## Supporting information


**Data S1:** jocd70578‐sup‐0001‐supinfo.docx.

## Data Availability

The data that support the findings of this study are available from the corresponding author upon reasonable request.
