# Supplementary material for: The Quality and Reliability of Short Videos About Melasma on TikTok and Bilibili: A Cross‐Sectional Study
Source: J Cosmet Dermatol. 2025 Dec 9;24(12):e70578. doi: 10.1111/jocd.70578 (PMC12687306; doi:10.1111/jocd.70578)
Supplement: Supplementary file 1 — Data S1: jocd70578‐sup‐0001‐supinfo.docx. [file JOCD-24-e70578-s001.docx]

**Example of a representative educational video (Etiology category)**

A dermatologist explained the common causes of melasma, emphasizing ultraviolet exposure, hormonal changes, and genetic susceptibility. The video used schematic diagrams to illustrate how melanin is produced and accumulated in the skin. The tone was educational and easy to understand for general viewers.
